# Hypotony maculopathy and photoreceptor folds with disruptions after vitrectomy for epiretinal membrane removal: two case reports

**DOI:** 10.1186/s13256-021-02824-3

**Published:** 2021-05-07

**Authors:** Yun Jeong Lee, Se Joon Woo

**Affiliations:** 1 Department of Ophthalmology, Seoul National University Hospital, Seoul, Republic of Korea; 2 Department of Ophthalmology, Seoul National University Bundang Hospital, Seoul National University College of Medicine, Seongnam, Republic of Korea

**Keywords:** Hypotony maculopathy, Pars plana vitrectomy, Epiretinal membrane

## Abstract

**Background:**

Hypotony maculopathy has been classically reported as a complication of glaucoma surgery or ocular trauma. There have been only a few reports of hypotony maculopathy following pars plana vitrectomy (PPV). Here, we report two cases of hypotony maculopathy occurring after PPV for epiretinal membrane (ERM) removal and characteristic photoreceptor folds observed on optical coherence tomography (OCT).

**Case presentation:**

A 53-year-old Korean woman (case 1) underwent phacoemulsification and posterior chamber lens implantation combined with 25-gauge PPV for ERM removal in the right eye. On the following day, she had severe ocular hypotony, with an intraocular pressure (IOP) that was unmeasurable using a pneumatic tonometer. Despite normalization of IOP, macular retinal and photoreceptor folds with photoreceptor disruptions developed, and Henle’s fiber layer hyperreflectivity was identified. Thereafter, retinal and photoreceptor folds gradually disappeared but photoreceptor disruption and Henle’s fiber layer hyperreflectivity did not improve until 1 year postoperatively, with persistent central visual field distortion and visual acuity worse than that at the preoperative state. A 20-year-old Korean man (case 2) underwent an additional 25-gauge PPV for ERM removal in the left eye. Examination on the following day showed ocular hypotony and retinal folds with peripheral choroidal detachment. Although IOP was normalized, further OCT revealed photoreceptor folds and photoreceptor disruptions. Since then, the photoreceptor folds resolved; however, the photoreceptor disruption remained in the macula at the 1-year follow up, with persistent distorted vision and visual acuity worse than that at the preoperative state.

**Conclusions:**

Early hypotony after vitrectomy for ERM could result in maculopathy leading to irreversible visual decline and metamorphopsia. Photoreceptor folds on OCT are characteristic features and the predominant mechanism of central visual loss in cases of hypotony maculopathy.

## Background

Hypotony maculopathy, which was first described by Dellaporta in 1954 [1] and denominated by Gass in 1972 [2], is a condition of low intraocular pressure (IOP) characterized by fundus abnormalities, including chorioretinal folds, optic disc edema, and vascular tortuosity [1]. In eyes with hypotony, the scleral wall collapses and the retina and choroid become redundant, which can result in chorioretinal folds and distortion of the photoreceptors [2, 3]. Most cases have been reported post-glaucoma surgery or ocular trauma, of which the incidence is known to range from 1.3% to 18% after glaucoma filtering surgery [4], with only a few reports concerning hypotony maculopathy following pars plana vitrectomy (PPV) [5, 6]. Herein, we report two cases of hypotony maculopathy occurring after PPV for epiretinal membrane (ERM) removal and characteristic photoreceptor folds identified by optical coherence tomography (OCT).

## Case presentation

### Case 1

**Fig. 1:**
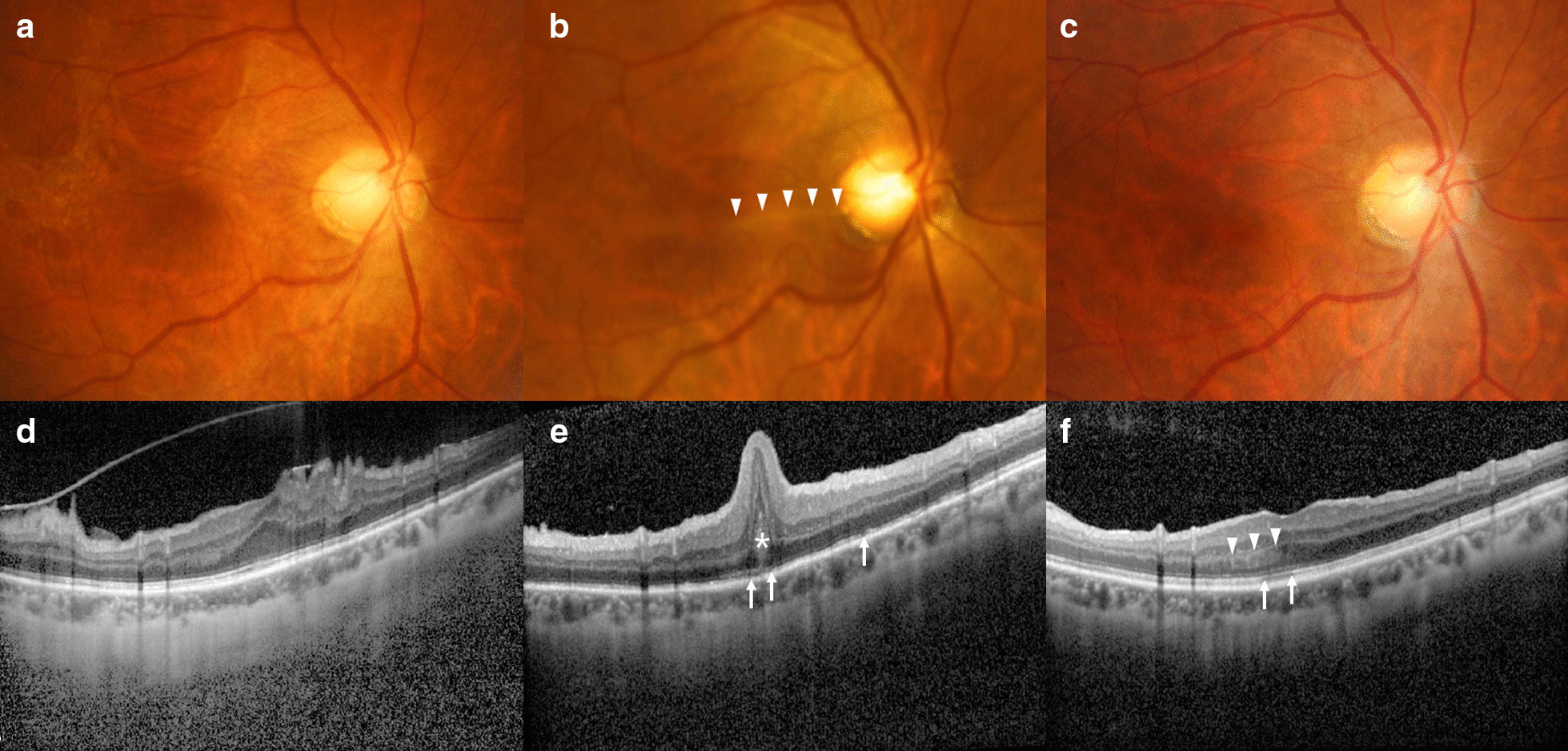
Fundus photography and optical coherence tomography (OCT) before and after the operation in case 1. **a** Fundus photography prior to surgery reveals an epiretinal membrane in the right eye. **b** Fundus photography 1 week after the operation shows macular retinal fold in the right eye (white arrowheads). **c** Fundus photography 1 year after the operation shows improved retinal fold in the right eye. **d** OCT of the macula before the operation shows epiretinal membrane with macular thickening in the right eye. **e** OCT of the macula 1 week after the operation demonstrates retinal and photoreceptor fold (stellate) with photoreceptor disruptions in the right eye (white arrows). **f** OCT of the macula 1 year after the operation demonstrates improved retinal and photoreceptor fold but remaining photoreceptor disruption (white arrows) and hyperreflectivity of Henle’s fiber layer (white arrowheads) in the right eye

A 53-year-old Korean woman visited our ophthalmology clinic for a thorough examination of retinal abnormalities which were identified during a regular checkup in both eyes, with a symptom of distorted vision (metamorphopsia) of the right eye of unknown onset. She had undergone laser-assisted *in situ* keratomileusis for myopia in both eyes at 26 years of age and had no prior history of glaucoma, family history of ocular disease, or psychosocial history. Her best-corrected visual acuity (BCVA) was 20/30 in the right eye and 20/16 in the left eye, and the manifest refractive error was −2.00Ds −0.25Dc × 010A in the right eye and −1.25Ds −0.75Dc × 085A in the left eye. Anterior segment examination showed nuclear sclerosis in both eyes. Further dilated fundus examination and OCT revealed ERM with retinal thickening (Fig. 1a, d). Following diagnosis of ERM and cataract in both eyes, she underwent phacoemulsification and posterior chamber lens implantation combined with transconjunctival sutureless 25-gauge PPV, ERM removal, and internal limiting membrane (ILM) peeling in the right eye. Vitreous was filled with balanced salt solution (BSS), and no other tamponade was performed. On the day after surgery, she had ocular hypotony in the right eye, with an IOP that was unmeasurable using a pneumatic tonometer, and no leakage was observed from sclerotomy sites. Since hypotony maculopathy can improve spontaneously over time with conservative management, we recommended that she keep using postoperatively prescribed eye drops including topical antibiotics, steroid, and cycloplegics rather than performing immediate surgical interventions. One week post-surgery, her BCVA was 20/500, and IOP improved to 7 mmHg, as measured by a pneumatic tonometer, in the right eye. Fundus photography and OCT, however, revealed macular retinal and photoreceptor folds with photoreceptor disruption (Fig. 1b, e). Thereafter, IOP remained stable throughout the entire follow-up period, which was 14 mmHg and 7 mmHg at 1 month and 8 months post-surgery, respectively. Also, the retinal and photoreceptor fold improved gradually along with BCVA, which was 20/100 and 20/40 at 1 month and 8 months post-surgery, respectively, although the patient presented a symptom of distortion of the central visual field in the right eye. At the final visit, 1 year post-surgery, she complained of persistent central visual field distortion, and her BCVA had improved to 20/40 but did not recover to the preoperative state. Further OCT revealed resolved photoreceptor folds but remnant mild photoreceptor disruption and hyperreflectivity of Henle’s fiber layer (Fig. 1c, f).

### Case 2

**Fig. 2:**
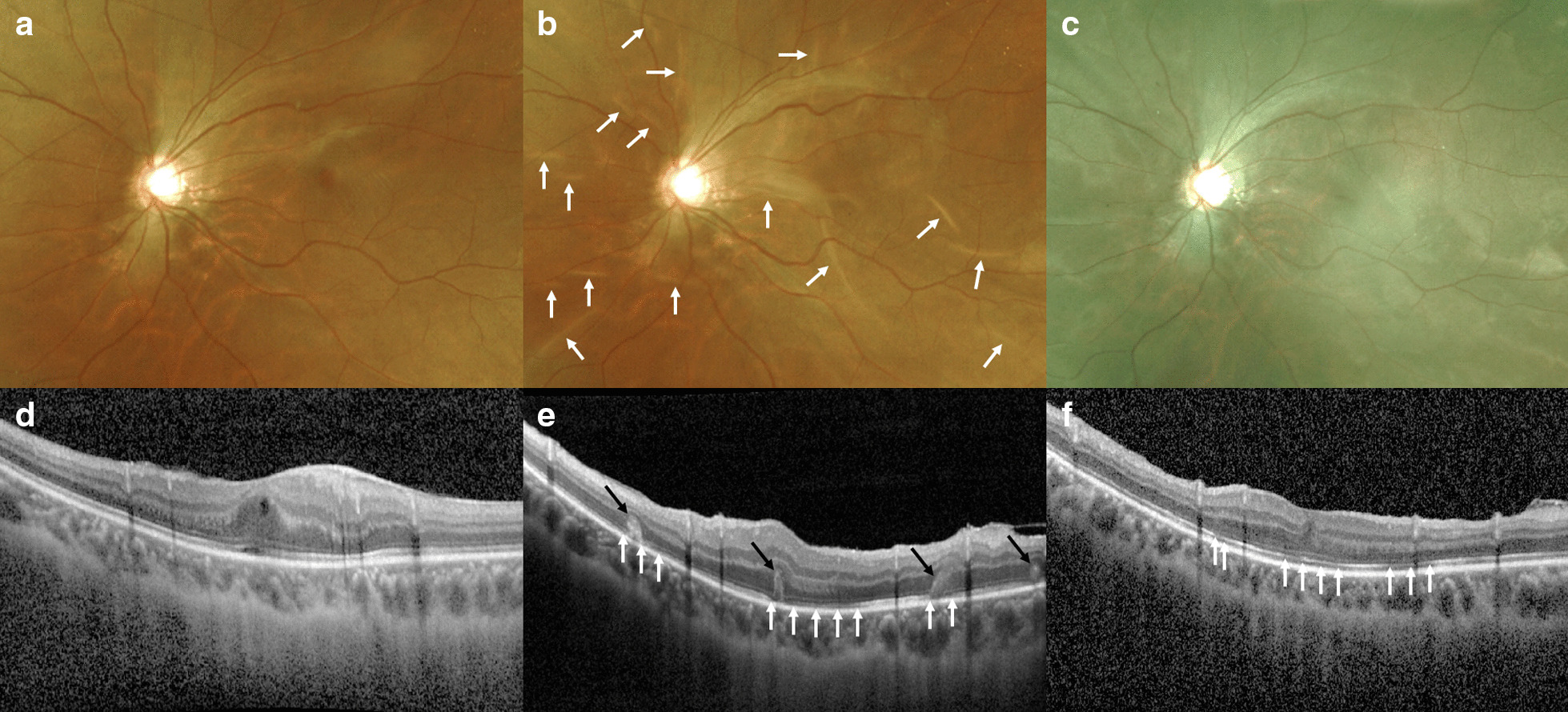
Fundus photography and optical coherence tomography (OCT) before and after the operation in case 2. **a** Fundus photography before the operation shows epiretinal membrane in the left eye. **b** Fundus photography 2 weeks after the operation shows multiple retinal folds including the macula in the left eye (white arrows). **c** Fundus photography 1 year after the operation shows improved retinal folds in the left eye. **d** OCT of the macula before the operation shows epiretinal membrane with macular edema in the left eye. **e** OCT of the macula 2 weeks after the operation demonstrates retinal and photoreceptor folds (black arrows) with photoreceptor disruptions in the left eye (white arrows). **f** OCT of the macula 1 year after the operation demonstrates improved retinal and photoreceptor folds but remaining photoreceptor disruptions in the left eye (white arrows)

A 20-year-old Korean man presented with distorted vision (metamorphopsia) of the left eye. He underwent combined cataract surgery and PPV in the left eye for cataract and rhegmatogenous retinal detachment at the age of 18 years and had no prior history of glaucoma, family history of ocular disease, or psychosocial history. On examination, his BCVA was 20/16 in the right eye and 20/40 in the left eye, and the manifest refractive error was −8.25Ds −1.50Dc × 180A in the right eye and −8.25Ds −0.25Dc × 065A in the left eye. Dilated fundus examination and OCT revealed macular ERM in the left eye (Fig 2a, d). He underwent additional transconjunctival sutureless 25-gauge PPV, ERM removal and ILM peeling without tamponade in the left eye. Examination on the following day showed ocular hypotony, with an IOP that was unmeasurable using a pneumatic tonometer, and multiple retinal folds involving the macula with choroidal detachment in the left eye. No leakage was found from sclerotomy sites. We recommended that he keep using eye drops including topical antibiotics, steroid, and cycloplegics, and additionally prescribed oral steroid to further help reduce inflammation. Examination 5 days post-surgery showed a normalized IOP of 19 mmHg using a pneumatic tonometer and decreased retinal folds. Two weeks post-surgery, his BCVA was 20/200 and IOP was 16 mmHg, with retinal folds gradually improving (Fig. 2b). OCT revealed multiple photoreceptor folds and disruptions (Fig. 2e). Thereafter, IOP remained stable during the entire follow-up period, which was 17 mmHg and 13 mmHg at 4 months and 8 months post-surgery, respectively, and the retinal and photoreceptor folds decreased. However, BCVA showed no improvement, which was 20/330 and 20/500 at 4 months and 8 months post-surgery, respectively. One year post-surgery, he complained of persistent distorted vision, and his BCVA was 20/500 in the left eye, which is worse than that at the preoperative state. Fundus examination showed no retinal folds, and OCT revealed mild photoreceptor disruptions in the macula (Fig. 2c, f).

## Discussion and conclusions

The treatment of hypotony maculopathy depends on the causes of hypotony and requires prompt management, as delayed normalization of IOP could give rise to permanent chorioretinal changes and result in poor visual outcomes [7, 8]. Fortunately, our patients’ IOP normalized spontaneously within a few days without the need for any further surgical treatment. Regarding the possible etiologies of hypotony in our patients, hypotony might be caused by sclerotomy site leakage that had resolved spontaneously, since no definite leakage was observed from sclerotomy sites on the day after the operation. Also, postoperative inflammation might have contributed to the development of hypotony, since it causes a reduction in aqueous production and increase in uveoscleral outflow. Analysis of the cause of hypotony maculopathy in our patients suggested that, firstly, the decrease in structural support due to retinal structural change during ERM removal and ILM peeling could have made the macular area more vulnerable to folding and distortion during the collapse of the eyeball. Second, both patients presented known risk factors for sclerotomy leakage and postoperative hypotony after transconjunctival sutureless vitrectomy, which are prior vitrectomy, young age (< 50 years), vitreous base dissection for sclerotomy leakage, myopia, and gas tamponade for early postoperative hypotony [9]. The patient in case 1 had one risk factor (myopia), and the patient in case 2 presented three risk factors (prior vitrectomy, young age, and myopia). Furthermore, both patients shared some of the other reported risk factors for hypotony maculopathy, which are young age (< 60 years), male sex, and myopia [4]. Among these risk factors, young age and myopia are associated with low scleral rigidity, which has been proposed as a crucial factor in the pathogenesis of hypotony maculopathy [4]. Also, the relationship of hypotony with gas tamponade was suggested in a previous report by Ahn *et al*. [10], where they proposed transient gas leakage through the sclerotomy site as a causative mechanism for postoperative 2-hour hypotony. Considering the susceptibility to developing hypotony maculopathy in the high-risk group after PPV, especially when performing ERM or ILM removal, additional procedures, such as suturing sclerotomy sites after surgery, to prevent leakage can aid in reducing the occurrence of hypotony maculopathy in these patients, though the procedures were not performed in our patients. Also, in cases where chorioretinal folds persist despite normalization of IOP, PPV, ILM peeling, and gas tamponade could be considered as one of the treatment options [3, 11, 12].

Visual acuity improved for the patient in case 1, whereas it did not recover for the patient in case 2, due to photoreceptor damage, despite normalization of IOP. The structural integrity of the macula and visual outcome are closely related; our cases share common features with those reported in a previous study by Ahn *et al*. [10], wherein morphological changes in the photoreceptor layer were observed after resolution of macular folds, which occurred after vitrectomy and gas tamponade injection for retinal detachment. Similar to a previous report [10], photoreceptor disruption was identified in both cases and Henle’s fiber layer hyperreflectivity in case 1. Moreover, it is noteworthy that characteristic photoreceptor folds were identified on early postoperative OCT under the retinal folds in both patients, which has not been previously reported. Our finding of photoreceptor folds is clinically important due to the photoreceptor damage arising from mechanical damage exerted during the photoreceptor fold formation, which can result in irreversible central vision loss. Although both patients showed photoreceptor disruption at the final visit, the difference in the final visual outcome between the two patients could be due to the difference in the amount and range of photoreceptor damage and potential eccentric fixation. Therefore, patients who have risk factors for sclerotomy leakage and hypotony such as prior vitrectomy and young age should be informed about the potential complications of hypotony maculopathy after ERM removal surgery and the possibility of poor visual outcome. Additionally, patients should be instructed to visit the clinic immediately in the case of visual symptoms such as blurred or distorted vision, which could be symptoms of hypotony maculopathy.

In conclusion, we report two cases of hypotony maculopathy after PPV for ERM removal and the hallmark of macular photoreceptor folds on OCT. This report will help in understanding the mechanism of hypotony maculopathy following PPV for ERM removal and in preventing this vision-threatening complication.

## Data Availability

All data in this case report are available from the corresponding author on reasonable request.

## Abbreviations

PPV: Pars plana vitrectomy
ERM: Epiretinal membrane
ILM: Internal limiting membrane
BCVA: Best-corrected visual acuity
OCT: Optical coherence tomography
IOP: Intraocular pressure
